# Advanced Biomaterials for Craniofacial Tissue Regeneration: From Fundamental Mechanism to Translational Applications—A Scoping Review

**DOI:** 10.3390/jfb17010044

**Published:** 2026-01-15

**Authors:** Żaneta Anna Mierzejewska, Valentina Veselinović, Nataša Trtić, Saša Marin, Jan Borys, Bożena Antonowicz

**Affiliations:** 1 Institute of Biomedical Engineering, Faculty of Mechanical Department, Bialystok University of Technology, Wiejska 45C, 15-351 Bialystok, Poland; 2 Faculty of Medicine, University of Banja Luka, 78000 Banja Luka, Bosnia and Herzegovina; valentina.veselinovic@med.unibl.org (V.V.); natasa.trtic@med.unibl.org (N.T.); sasa.marin@med.unibl.org (S.M.); 3 Department of Maxillofacial and Plastic Surgery, Medical University of Bialystok, M. Sklodowskiej-Curie 24A, 15-276 Bialystok, Poland; jan.borys@umb.edu.pl; 4 Department of Dental Surgery, Medical University of Bialystok, M. Sklodowskiej-Curie 24A, 15-276 Bialystok, Poland; bozena.antonowicz@umb.edu.pl

**Keywords:** biomaterials, tissue engineering, maxillofacial reconstruction, stem cell therapy, 3D bioprinting

## Abstract

Recent advances in biomaterials, immunomodulation, stem cell therapy, and biofabrication are reshaping maxillofacial surgery, shifting reconstruction paradigms toward biologically integrated and patient-specific tissue regeneration. This review provides a comprehensive synthesis of current and emerging strategies for bone and soft-tissue regeneration in the craniofacial region, with particular emphasis on bioactive ceramics, biodegradable polymers, hybrid composites, and stimuli-responsive smart materials. We further examine translational technologies such as extracellular vesicles, decellularized extracellular matrices, organoids, and 3D bioprinting, highlighting key challenges such as bioink standardization, perfusion limitations, and regulatory classification. Maxillofacial surgery is positioned for a paradigm shift toward personalized, biologically active, and clinically scalable regenerative solutions.

## 1. Introduction

**Figure 1:**
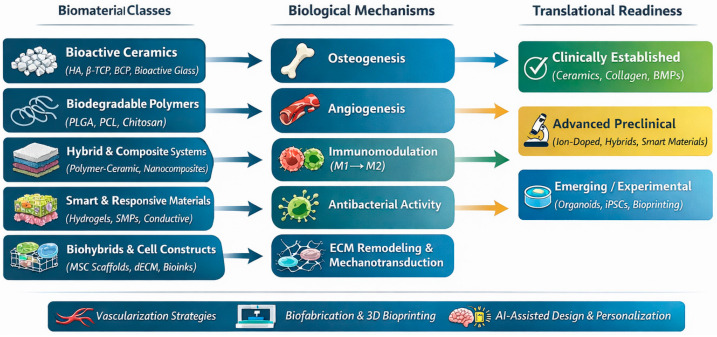
Integrated framework of advanced biomaterials for craniofacial tissue regeneration. The schematic illustrates the interconnections between major biomaterial classes used in craniofacial regeneration (**left**), their primary biological mechanisms of action (**center**), and their current level of translational readiness (**right**). Bioactive ceramics, biodegradable polymers, hybrid composites, smart materials, and biohybrid constructs modulate key regenerative pathways, including osteogenesis, angiogenesis, immunomodulation, antibacterial activity, and extracellular matrix remodeling. The translational spectrum highlights the disparity between clinically established materials and emerging experimental strategies such as organoids and bioprinted constructs. Cross-cutting factors, including vascularization strategies, biofabrication technologies, AI-assisted design, and regulatory constraints, influence successful clinical translation across all material classes.

Reconstruction of craniofacial defects arising from trauma, tumor resections, infections, or congenital anomalies remains a major challenge in maxillofacial surgery [1]. Traditional approaches rely heavily on autografts, which offer osteogenic, osteoconductive, and osteoinductive potential but are limited by donor-site morbidity, limited supply, and unpredictable long-term stability in large defects [2]. Allografts and xenografts improve availability but introduce risks of immune reaction, variable resorption, and pathogen transmission [3,4]. Over the last decade, rapid growth in biomaterials science and biotechnology has transformed maxillofacial regenerative strategies. Developments in bioactive ceramics, polymer–ceramic composites, hydrogels, and 3D-printed scaffolds have significantly improved the ability to mimic natural tissue architecture and modulate the healing microenvironment [5,6]. Parallel advances in controlled drug delivery, stem cell therapy, extracellular vesicle biology, and smart materials have further expanded the therapeutic toolkit, enabling targeted regulation of osteogenesis, angiogenesis, and immune responses [7,8]. Many advanced biomaterials perform well in preclinical studies but fail to meet regulatory, manufacturing, or biological requirements for clinical implementation. Despite significant advances, the field remains fragmented across material classes, biological mechanisms, and translational stages. This review integrates the fields of biomaterials science, biology of regeneration, cellular therapy, and emerging biofabrication technologies to provide a coherent framework for future translational progress in maxillofacial reconstruction (Figure 1).

Despite the growing number of reviews addressing individual classes of biomaterials, specific fabrication technologies, or selected biological mechanisms, an integrative perspective linking material design, biological function, and translational readiness in craniofacial regeneration remains limited. Many existing reviews focus either on material chemistry or on isolated regenerative strategies without systematically addressing their clinical maturity, regulatory constraints, and scalability. The present review addresses this gap by providing a unified framework that connects advanced biomaterial classes with their underlying biological mechanisms and translational challenges, thereby offering guidance for the development of clinically viable, next-generation regenerative solutions for the craniofacial region. Accordingly, this work presents an integrative narrative review that synthesizes preclinical and clinical evidence on advanced biomaterials for craniofacial tissue regeneration with a specific focus on translational applicability.

## 2. Review Methodology and Search Strategy

**Figure 2:**
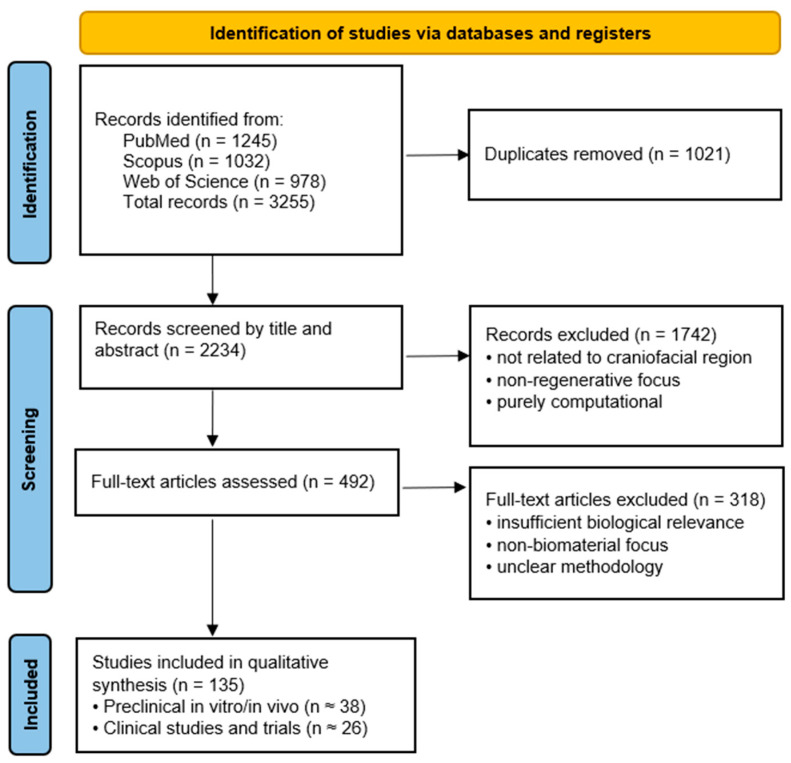
Literature selection flow illustrating the identification, screening, and inclusion of sources reviewed in this study.

The review protocol was registered in the Open Science Framework (OSF; registration number: 10.17605/OSF.IO/PC5UE). This review was designed as an integrative narrative synthesis aimed at mapping current advances in biomaterials for craniofacial tissue regeneration and highlighting their biological mechanisms and translational readiness. The literature identification and selection process is summarized in Figure 2. Owing to the heterogeneity of biomaterials, experimental models, and outcome measures across the included studies, a quantitative meta-analysis was not feasible. Instead, studies were categorized according to material class, biological mechanism, and translational stage (preclinical vs. clinical), allowing qualitative synthesis and identification of translational bottlenecks.

The objective was to summarize current and emerging biomaterials, cellular therapies, and biofabrication technologies relevant to maxillofacial tissue regeneration. A comprehensive literature search was performed in PubMed, Scopus, and Web of Science for articles published between January 2020 and November 2025. The following keyword combinations were used: maxillofacial regeneration, biomaterials, tissue engineering, bioactive ceramics, polymer scaffolds, growth factor delivery, exosomes, 3D bioprinting, and smart materials. Additional articles were identified through reference screening of relevant reviews and clinical reports.

Inclusion criteria comprised: (1) studies involving biomaterials or regenerative strategies applicable to maxillofacial surgery; (2) in vitro, in vivo, or clinical research; and (3) peer-reviewed original papers or reviews. Exclusion criteria included: (1) non-biomedical engineering materials, (2) purely computational studies without biological relevance, and (3) articles lacking methodological clarity.

## 3. Biomaterials Strategies for Maxillofacial Regeneration

### 3.1. Bioactive Ceramics and Composites

Bioactive ceramics remain a cornerstone of bone tissue engineering in maxillofacial surgery, owing to their excellent biocompatibility, structural stability, and capacity to promote osteogenesis through direct chemical bonding with bone. The most widely studied materials include hydroxyapatite (HA), which mimics the inorganic mineral phase of bone, characterized by slow degradation and long-term stability, making it suitable for regions requiring sustained mechanical support β-tricalcium phosphate (β-TCP), known for its predictable biodegradation, exhibits a higher resorption rate, allowing gradual replacement by newly formed bone and supporting natural remodeling without leaving permanent foreign material [9]. Biphasic calcium phosphates (BCPs), combining HA and β-TCP, provide an optimized balance between stability and controlled resorption, frequently outperforming single-component materials in clinical and histological studies [10]. And bioactive glasses (BAG), capable of forming a hydroxycarbonate apatite layer on their surface [11]. These materials are valued for their clinical utility spans alveolar ridge augmentation, sinus floor elevation, and reconstruction of small-to-moderate defects.

#### Bioactive Ion Doping in Ceramics for Bone Regeneration

A highly promising strategy for enhancing the biological activity of HA, β-TCP, and BAG involves incorporating therapeutic ions such as strontium (Sr^2+^), silicon (Si^4+^), and zinc (Zn^2+^) into their crystalline structure.

In vitro and in vivo studies have demonstrated that substituting strontium ions (Sr^2+^) into the structure of HA or BAG significantly enhances osteoregenerative processes [12,13]. Strontium ions stimulate the proliferation and differentiation of osteoblasts, increasing the expression of osteogenic markers such as osteocalcin, alkaline phosphatase (ALP), and type I collagen [14]. At the same time, Sr^2+^ inhibits osteoclast activity, reducing bone resorption and improving overall bone balance. This effect is partly mediated through modulation of the RANK/RANKL/OPG pathway, in which strontium upregulates osteoprotegerin (OPG), an antagonist of RANKL, thereby suppressing osteoclast maturation and activity [15,16]. In animal models, Sr-HA and Sr-BAG implants exhibit superior bone integration, increased regenerated bone volume, and higher hardness and elastic modulus compared to their strontium-free counterparts [17,18,19]. Synergistic effects when combined with ions such as Zn^2+^ or Mg^2+^ have also been reported, promoting angiogenesis and modulating inflammatory responses [20].

Silicon ions (Si^4+^) play a crucial role in bone regeneration. Si^4+^ initiates rapid formation of a hydroxyapatite layer on the surface of the biomaterial. This mechanism involves the release of silicon ions, which promote the creation of a local environment favorable for apatite nucleation and crystallization, also modulate the expression of osteogenic genes [21]. Experimental studies have shown that Si^4+^ stimulates the upregulation of osteoblastic markers, including osteocalcin, type I collagen, ALP, and Runx2, leading to accelerated osteoblast differentiation and mineralization [22,23]. The release of Si^4+^ from bioactive glasses promotes angiogenesis through increased expression of vascular endothelial growth factor [24]. Silicon ions also exhibit immunomodulatory properties, reducing chronic inflammatory responses at the implantation site, which favors stable integration of the biomaterial with the host tissue [25].

Zinc (Zn^2+^) is a trace element essential for numerous enzymatic processes involved in bone formation and regeneration. It participates in the activity of enzymes such as ALP, collagenase, and matrix metalloproteinases, which are critical for collagen synthesis and mineralization of the bone matrix [26,27]. In vivo studies have shown that ceramic implants containing Zn^2+^ demonstrate improved bone integration and reduced bacterial colonization compared to zinc-free counterparts [28]—Zn^2+^ exhibits antibacterial properties, inhibiting the growth of both Gram-positive and Gram-negative bacteria. This is particularly important in the context of biomaterial implantation, where the risk of infection is one of the most common complications in reconstructive surgery. The mechanism involves induction of oxidative stress in bacterial cells and disruption of essential enzymatic functions necessary for their survival [29]. Moreover, zinc can act synergistically with other bioactive ions, such as Si^4+^ or Sr^2+^, enhancing both osteogenic processes and the antibacterial properties of the biomaterial [30].

Because of these characteristics, calcium-phosphate ceramics and bioactive glasses enriched with Sr^2+^, Si^4+^ or Zn^2+^ are particularly useful in regenerative dentistry (alveolar ridge augmentation, sinus reconstruction), orthopedics (bone defect fillers, structural implants), and tissue engineering, where combining bioactivity, osteogenic stimulation, and angiogenic support is essential.

### 3.2. Biodegradable Polymers in Craniofacial Regeneration

Biodegradable polymers constitute an essential class of biomaterials in contemporary tissue engineering, particularly in maxillofacial surgery, where they serve as versatile scaffolds capable of supporting three-dimensional tissue growth, controlled drug release, and cell integration. Their popularity stems from the ability to fine-tune mechanical properties, degradation kinetics, porosity, and biological functionality, allowing the design of scaffolds suited to the specific architecture of bone defects and the biological requirements of regenerating tissues [31,32]. Among the polymers most widely used in maxillofacial reconstruction are poly(lactic-co-glycolic acid) (PLGA), poly-ε-caprolactone (PCL), and chitosan. Each material exhibits distinct properties that determine its clinical applications, and recent developments increasingly focus on combining them with bioactive agents, nanoparticles, or ceramics to achieve multifunctional hybrid scaffolds [33].

PLGA is a biodegradable copolymer, degrades via hydrolysis of ester bonds, releasing lactic and glycolic acid—both naturally metabolized by the body [34]. PLGA has been extensively studied as a carrier for growth factors, and various antimicrobial or anti-inflammatory agents. Its ability to encapsulate and gradually release bioactive molecules makes it a powerful tool for engineered osteoinduction and angiogenesis. Numerous in vivo studies demonstrate that PLGA-based BMP-2 delivery systems increase bone mineral density, accelerate defect closure, and enhance implant integration while reducing complications associated with high single-dose BMP-2 administration [35,36,37]. PLGA is also widely used as a platform for microspheres and nanocarriers, enabling temporally controlled release profiles and co-delivery strategies essential for large, poorly vascularized defects typical of the maxillofacial region.

Poly-ε-caprolactone (PCL) is a partially crystalline, thermoplastic polymer with a long degradation time, extending up to several years, making it particularly useful for large defects requiring sustained mechanical support, such as segmental mandibular reconstruction or vertical alveolar ridge augmentation. Due to its low melting temperature and viscoelastic behavior, PCL is highly compatible with 3D printing technologies, enabling precise control of scaffold geometry, pore size, and mechanical anisotropy [38]. Studies have shown that PCL implants support osteoblast migration, vascular ingrowth, and bone matrix mineralization, promoting effective osteoconduction. Combined with bioactive additives, such as hydroxyapatite or bioactive glasses, PCL exhibits enhanced bioactivity and improved mechanical scaffold properties, as confirmed in both animal models and preclinical studies [39,40].

Chitosan is valued for its inherent biocompatibility, biodegradability, hemostatic activity, and intrinsic antibacterial properties. Its chemical structure—with abundant amino groups—allows versatile functionalization, enabling the fabrication of hydrogels, nanofibers, injectable pastes, and polymer–ceramic composites that mimic the extracellular matrix (ECM) [41]. Chitosan-based scaffolds support osteoblast adhesion, proliferation, and early mineralization, while also promoting angiogenesis and reducing bacterial colonization. Importantly, chitosan can be blended with PCL, PLGA, or HA to improve mechanical stability and create hybrid structures suited for complex maxillofacial defects, where standalone natural polymers would be insufficient [42,43].

#### Hybrid Polymer Systems for Controlled Bioactivity and Cell Delivery

Polymer-based scaffolds have evolved from passive frameworks into active bioreactors that not only support cellular growth but also modulate the microenvironment of regenerating tissue at the defect site. A major frontier in polymer-based regenerative strategies is the development of multifunctional hybrid systems that integrate controlled release of bioactive factors with support for cell-based therapies. PLGA and PCL are highly effective carriers for osteogenic and angiogenic molecules [44,45]. Controlled and sustained release of BMP-2 from PLGA microspheres incorporated into a scaffold matrix enhances the rate and quality of bone formation more effectively than single-dose bolus administration [46,47]. By maintaining a stable therapeutic concentration of BMP-2 within the microenvironment, these systems ensure continuous stimulation of osteoprogenitor cells. Moreover, integrating polymers with other bioactive agents—such as vascular endothelial growth factor (VEGF)—has been shown to synergistically stimulate osteogenesis and angiogenesis [48].

Biodegradable scaffolds also provide a supportive three-dimensional niche for mesenchymal stem cells (MSCs), adipose-derived stem cells (ADSCs), and dental pulp stem cells (DPSCs). Proper pore architecture and surface chemistry of scaffolds promote high viability, proliferation, and osteogenic differentiation of seeded cells [36]. In animal models, PLGA and PCL scaffolds seeded with BMSC led to faster bone defect regeneration and greater mineralization compared to control scaffolds without cells. Furthermore, combinations of polymer scaffolds and stem cells significantly increased the expression of osteogenic genes, correlating with robust bone matrix deposition and faster restoration of mechanical function in the regenerated area [14,38,48].

The most advanced designs combine both strategies—cell support and controlled release of bioactive factors—within a single polymeric system. These constructs act as bioactive bioreactors, simultaneously providing mechanical stability, spatial guidance, and biochemical stimulation. Preclinical and early clinical studies indicate that such systems significantly accelerate the regeneration of large bone defects in the maxilla and mandible and improve the quality of newly formed tissue through enhanced vascularization and mineralization [49,50].

### 3.3. Biohybrids and Smart Materials for Tissue Regeneration

Smart biomaterials represent a new generation of regenerative platforms capable of actively modulating the local microenvironment in response to mechanical, biochemical, or physical stimuli. Unlike conventional scaffolds, which act primarily as passive structural supports, smart materials participate in the healing process by releasing therapeutic agents, altering stiffness, or adjusting degradation profiles in real time. This dynamic behavior is particularly important in maxillofacial surgery, where tissue regeneration occurs in environments characterized by complex loading patterns, microbial exposure, and localized inflammatory responses [51]. Several classes of smart biomaterials have gained attention in recent years, including stimuli-responsive hydrogels, shape-memory polymers, self-healing materials, magneto-responsive nanocomposites, and photoactivated systems.

#### Responsive Materials Mechanisms

Stimuli-responsive hydrogels are polymer networks capable of undergoing reversible physicochemical changes in response to pH, temperature, enzymatic activity, or external stimuli such as light or ultrasound. In maxillofacial reconstruction, they offer several advantages: they can be injected minimally invasively, conform to irregular bone defects, and serve as carriers for growth factors, stem cells, peptides, and extracellular vesicles [52]. pH-responsive hydrogels have been engineered to release antimicrobial agents or anti-inflammatory drugs in acidic environments characteristic of early inflammation or infection. Such systems may reduce complications associated with bone graft contamination, which remains a notable concern in oral surgery [53]. Thermo-responsive hydrogels, such as those based on modified chitosan, remain fluid at room temperature but gel upon reaching body temperature, enabling controlled in situ scaffold formation. When combined with MSCs or DPSCs, they improve cell viability and retention, accelerating bone deposition in alveolar and periodontal defects [54]. Enzyme-responsive hydrogels that degrade in response to matrix metalloproteinases (MMPs) or ALP allow synchronized scaffold remodeling with bone formation. This makes them highly suitable for regeneration in dynamic mechanical environments of the mandible and maxilla [55].

Shape-memory polymers (SMPs) can recover a pre-defined geometry when exposed to an external trigger such as temperature, moisture, or light. In maxillofacial surgery, SMPs are particularly valuable for minimally invasive insertion into complex anatomical spaces. After placement, they expand or change shape to restore structural and mechanical stability, reduces micromovement at the healing site—an important factor for predictable bone integration and provide intimate contact with surrounding tissues [56].

Self-healing biomaterials contain reversible chemical motifs—such as Schiff-base bonds, dynamic covalent linkages, or supramolecular interactions—that allow the scaffold to repair microcracks without external intervention. This property is particularly relevant for maxillofacial defects subjected to repeated mastication forces and cyclic loading. Self-healing hydrogels based on chitosan, or gelatin, derivatives improve long-term structural integrity while maintaining high water content and biocompatibility. In vivo studies suggest that self-healing polymer composites loaded with osteogenic nanoparticles or growth factors enhance defect stability and reduce premature scaffold failure, promoting consistent bone regeneration [57,58,59].

Both electromagnetic and magnetically responsive materials offer unique opportunities to modulate cell behavior during regeneration. Magneto-responsive scaffolds, incorporating iron oxide nanoparticles, can provide localized mechanical cues under an external magnetic field, stimulating osteoblast differentiation and enhancing mineral deposition. They also enable targeted drug delivery, directing therapeutic nanoparticles to specific anatomical regions within the maxillofacial skeleton [60]. Electro-conductive scaffolds, based on polypyrrole, polyaniline, or graphene derivatives, mimic the endogenous electrical signals involved in bone remodeling. Electrical conductivity enhances osteogenic differentiation of MSCs and promotes vascularization VEGF pathway [61]. Such scaffolds are especially promising for large or critical-sized defects where biological signaling needs reinforcement.

Light-triggered biomaterials allow precise spatial and temporal control of scaffold behavior. UV—or near-infrared-responsive materials can activate drug release, trigger crosslinking, or modulate stiffness on demand. Photo-crosslinkable hydrogels such as GelMA (gelatin methacryloyl) are widely used as bioinks for 3D bioprinting due to their tunable mechanics, high cytocompatibility, and ability to encapsulate cells in a supportive matrix [62]. When used in maxillofacial reconstruction, GelMA-based constructs enhance osteogenic differentiation, integrate well with host bone, and support vascular infiltration. Light-responsive nanoparticles embedded in polymer matrices can also activate antimicrobial activity or promote localized hyperthermia, providing additional protection in oral environments with high microbial load [63,64]. A summary of the key biomaterials used in maxillofacial reconstruction, including their properties and principal clinical applications, is presented in Table 1.

## 4. Functionalization and Surface Engineering

### 4.1. Scaffolds Architecture and Surface Engineering

Beyond chemical composition, the architecture of the material plays a fundamental role in regenerative success. This porous network facilitates vascular infiltration, ensuring the transport of nutrients, oxygen, and ions necessary for mineralization, while allowing removal of metabolic waste, which is critical for the proper function of rapidly growing bone tissue [65]. The porous architecture also serves as a scaffold for osteoconduction, guiding the directional growth of new bone tissue within which effective regeneration and matrix mineralization occur [66].

Modern fabrication techniques such as 3D printing, freeze-casting, and controlled-porosity sintering enable the creation of gradient pore structures, tailored to specific anatomical defects. Customized ceramic scaffolds produced with additive manufacturing allow precise modulation of mechanical strength, porosity, stiffness, and geometry, which can be optimized for load distribution in mandibular and maxillary reconstructions. Architectural optimization increasingly includes hierarchical designs that integrate macro-porous frameworks with micro- and nano-scale topographical features that enhance protein adsorption, osteoblast adhesion, and initial cell signaling—bringing ceramic scaffolds closer to the complexity of natural bone [67].

Nanoscale architecture of polymer scaffolds represent a crucial aspect of contemporary tissue engineering strategies, as the interface between the material and the cell ultimately determines the success of tissue integration and regeneration. Properly engineered surface features at the nano- and microscale significantly influence the adhesion, proliferation, and differentiation of osteogenic cells, thereby defining the effectiveness of osteointegration, a topic discussed in detail in Section 6.2.1.

One of the most effective approaches for designing scaffolds with controlled architecture is electrospinning, a technique that enables the fabrication of fibers with diameters in the range of hundreds of nanometers, closely resembling the structure of ECM [68]. Such scaffolds exhibit a high surface-to-volume ratio, high porosity, and excellent permeability to nutrients, which promote cell migration and colonization. The nanoscale topography generated through electrospinning mimics the native biological environment, resulting in stronger osteoblastic adhesion and upregulated expression of osteogenic genes such as RUNX2, ALP, and OCN [69]. Experimental studies have shown that PCL and PLGA nanofibers with diameters below 500 nm significantly increase alkaline phosphatase activity and matrix mineralization compared to microscale scaffolds. Moreover, electrospun matrices can serve as intelligent delivery systems for bioactive agents—their surface or internal structure can be modified to incorporate growth factors, anti-inflammatory drugs, or mineral nanoparticles, enabling a synergistic effect between osteoconduction and osteoinduction [70].

### 4.2. Surface Functionalization and Nanoparticle Integration

Another important research direction involves the functional modification of polymer scaffolds. The deposition of thin layers of hydroxyapatite, bioactive glasses, or adhesive peptides represents a hybridization strategy that combines the flexibility and processability of polymers with the bioactivity of inorganic materials [34,39]. Such modifications promote immediate cellular recognition of the biomaterial, accelerating osteointegration. Studies have shown that PCL scaffolds coated with hydroxyapatite via electrochemical deposition or biomimetic precipitation significantly increase the expression of osteogenic markers, including alkaline phosphatase and osteocalcin, within the first days of cell culture [38,47]. Similarly, introducing adhesive peptides containing the RGD (arginine–glycine–aspartic acid) sequence—essential for integrin-mediated cell adhesion—onto the polymer surface enhances osteoblast proliferation and matrix mineralization. In vivo experiments have demonstrated that polymer implants functionalized with RGD or hydroxyapatite nanoparticles show superior bone integration and faster tissue formation compared to unmodified materials [71].

Advanced surface and nanoscale architectural modifications make it possible to design biomaterials with multilevel functionality, which not only provide appropriate mechanical properties and spatial scaffold architecture but also actively regulate cellular behavior through mechanical, topographical, and chemical cues. Emerging technologies such as 3D printing with nanocomposites, layer-by-layer self-assembled nanocoatings, and plasma surface modification allow precise control over both the chemical composition and the nanostructure of the material. This enables the creation of personalized implants with bioactivity tailored to individual patient needs—implants that not only fill bone defects but also actively guide regenerative processes [72,73].

Another rapidly developing direction in biomaterials engineering is the biofunctionalization of polymer scaffold surfaces through the incorporation of metal nanoparticles with bioactive and antimicrobial properties. This approach combines biological functionality with clinical safety, addressing one of the main challenges in modern reconstructive surgery—the high risk of postoperative infections and the need to accelerate osteointegration [74]. Metal nanoparticles such as Ag, Zn, and Cu, owing to their unique physicochemical and biological properties, provide simultaneous antibacterial, angiogenic, and osteogenic effects, making them particularly attractive additives to polymer-based regenerative systems [75].

The mechanism of AgNP action involves interaction with microbial cell membranes, generation of reactive oxygen species (ROS), and denaturation of proteins and nucleic acids, effectively eliminating both Gram-positive and Gram-negative bacteria, including resistant strains [76]. Numerous studies have shown that introducing small amounts of silver nanoparticles into polymer matrices can significantly reduce the risk of post-implantation infections without adversely affecting osteoblast proliferation. Moreover, low concentrations of AgNPs have been found to stimulate the expression of genes related to bone formation and vascularization, further supporting tissue regeneration [77,78,79].

Magnesium, introduced as nanoparticles or Mg^2+^ ions within the scaffold structure, plays a dual role—as a biologically active element regulating bone metabolism and as an antimicrobial agent. Studies have demonstrated that scaffolds modified with Mg^2+^ ions exhibit increased expression of osteogenic markers, along with enhanced surface mineralization in osteoblast cultures [20,40,80].

Copper nanoparticles (CuNPs), in turn, are distinguished by their strong proangiogenic activity, resulting from their ability to induce VEGF expression and stimulate the formation of new blood vessels [81]. Controlled amounts of CuNPs also enhance osteoblast differentiation and accelerate matrix mineralization. Polymer composites containing copper have been shown to promote faster bone formation and higher mineral density in animal models without inducing systemic toxicity [82].

The use of metallic nanoparticles in the biofunctionalization of polymer scaffolds represents one of the most promising avenues in the development of next-generation biomaterials. Such solutions not only enhance clinical safety by reducing infection risk but also actively promote bone and vessel regeneration, leading to faster and more integrated healing. In the context of maxillofacial surgery, where the implantation environment is often exposed to bacterial contamination and regeneration requires both mechanical and biological support, this approach paves the way for the creation of intelligent, personalized implant systems with triple action—osteogenic, angiogenic, and antibacterial. Combining polymer matrices with metal nanoparticles thus creates multifunctional systems that are mechanically stable, biologically active, and resistant to bacterial colonization. However, the control of metal ion concentration and release kinetics is crucial to maintaining the balance between biological activity and cytotoxicity [83].

### 4.3. Three-Dimensional Scaffolds and Growth Factor Signaling

The fundamental role of a scaffold is to provide both structural support and cellular guidance. The material must possess adequate mechanical strength to withstand the functional loads in the maxillofacial region, but most importantly, its porosity and interconnected pore network create a space conducive to the migration and ingrowth of stem cells, fibroblasts, and blood vessels, thereby enabling effective vascularization. Efficient perfusion is essential for cell survival and the formation of new, living tissue, which in dentistry directly impacts implant stability and the reconstruction of the alveolar ridge [4,49].

The scaffold also functions as a cell niche, delivering precisely orchestrated biochemical and mechanical signals. An ideal scaffold not only provides mechanical support but also directs cellular differentiation and function. It has been demonstrated that scaffold stiffness, measured by the Young’s modulus, directly influences stem cell fate, determining whether MSCs differentiate into osteoblasts, chondrocytes, or adipocytes [15,23,68]. This enables precise control over the regeneration process, which in clinical practice allows for the optimization of alveolar bone, periodontal, and oral soft tissue reconstruction. Thus, the scaffold becomes not merely a physical filler for the defect but an active tool that orchestrates the healing process, integrates with implanted stem cells, and supports the formation of functional, vascularized bone tissue [84].

Growth factors are potent peptide-based signaling proteins that serve as key regulators of biological processes, including cell proliferation, migration, and differentiation. In tissue engineering, they function as precise mediators of intercellular communication, initiating and coordinating tissue regeneration. For the repair process to proceed effectively, these factors must be delivered in a controlled manner—most commonly through their incorporation into the scaffold structure, for example, by adsorption or encapsulation, or in combination with cells at the time of implantation [85].

In the context of bone regeneration within the stomatognathic system, the most crucial growth factors include BMPs, VEGF, and Transforming Growth Factor β (TGF-β). Among them, BMP-2 and BMP-7 are some of the most potent osteoinductive agents known, capable of inducing mesenchymal stem cells to differentiate into osteoblasts, even in areas where bone formation does not naturally occur [86]. In dentistry, their use—particularly in combination with biocompatible carriers such as collagen, PLGA, or hydrogels—is now considered a standard approach in the treatment of large alveolar bone defects, as well as in reconstructions following tumor resections or sinus augmentation procedures [87].

At the same time, VEGF plays a fundamental role in angiogenesis, the formation of new blood vessels within regenerating tissue. Without adequate vascularization, oxygen and nutrients cannot reach the central regions of the scaffold, leading to necrosis and regeneration failure. Studies have shown that VEGF-releasing implant materials significantly accelerate vascularization and, consequently, enhance both the rate and quality of new bone tissue formation [88]. Meanwhile, TGF-β acts as a regulatory factor, supporting cell differentiation and stimulating the synthesis of ECM components, thereby promoting stabilization of the newly formed tissue [89,90].

A crucial aspect determining the success of growth factor–based therapies is the control of their release kinetics. An excessively rapid release—the so-called burst release—results in inefficiency and only short-term biological activity [91]. Therefore, modern scaffolds are designed as systems for controlled and sustained release, maintaining the growth factor concentration within the optimal therapeutic range throughout the critical healing period. Contemporary research in regenerative dentistry highlights that the synergy between a scaffold’s personalized architecture, its mechanical properties, and its capacity for targeted growth factor delivery forms the foundation of effective craniofacial bone regeneration.

## 5. Cellular Therapies, Organoids, and 3D Bioprinting

### 5.1. Cellular Approaches in Craniofacial Tissue Engineering

Tissue engineering is an interdisciplinary field aimed at creating biological substitutes capable of restoring, maintaining, or enhancing the function of damaged tissues. In dentistry, its applications focus primarily on the regeneration of hard and soft oral tissues, with particular emphasis on the reconstruction of alveolar bone defects, sinus floor augmentation, and the repair of periodontal defects. Key strategies include the use of stem or progenitor cells, which can differentiate along the osteogenic lineage; three-dimensional scaffolds that provide mechanical support and guidance for newly forming tissue; and growth factors that stimulate cell proliferation, migration, and differentiation. The integration of these elements creates an environment conducive to functional bone tissue regeneration, closely mimicking the natural process of osteogenesis [92].

#### 5.1.1. Advanced Stem-Cell Therapies

The use of stem cells represents one of the most revolutionary directions in regenerative medicine, offering the possibility of biologically restoring damaged tissues rather than simply replacing them. In the context of dentistry and maxillofacial surgery, cells capable of self-renewal and differentiation play a fundamental role, becoming a key element of tissue engineering strategies. Of particular importance are MSCs, which are currently the most intensively studied and promising source of cells for reconstructive therapies. Their appeal lies in their multipotency—the ability to differentiate into mesenchymal-derived cells, including osteoblasts, chondrocytes, adipocytes, and cementoblasts—enabling simultaneous regeneration of bone, cartilage, adipose tissue, and root cementum. Additionally, MSCs exhibit immunomodulatory properties, which help mitigate local inflammation and support healing following surgical procedures [93].

In dentistry and maxillofacial surgery, MSCs can be obtained from multiple, relatively accessible sources, which is crucial for clinical practice. Traditionally, bone marrow-derived cells have been used; however, increasing attention is being paid to cells derived from oral tissues. An example is DPSCs, isolated from extracted teeth, which exhibit high proliferative potential as well as the ability to differentiate into osteogenic and odontogenic lineages. This makes them particularly valuable for regenerating alveolar bone and the tooth–periodontal complex, and in vivo studies have demonstrated their effectiveness in forming vascularized and mineralized bone [94,95]. Another important source is periodontal ligament stem cells (PDLSCs), which play a key role in regenerating periodontal structures, including cementum, alveolar bone, and the ligament itself, essential for treating advanced periodontal diseases [96]. Additionally, adipose-derived stem cells (ADSCs) obtained from fat tissue provide an attractive alternative to bone marrow MSCs, as they are easier to harvest in larger quantities, and their osteogenic potential is comparable, making them a promising material for filling larger bone defects in the jaw [97,98].

At a more advanced research level, induced pluripotent stem cells (iPSCs) are being investigated. These cells are generated by genetically reprogramming somatic cells, such as skin fibroblasts, to a pluripotent state. iPSCs offer the possibility of producing autologous cell populations functionally equivalent to embryonic stem cells, thereby avoiding the ethical concerns associated with ESCs. Although their clinical use in dentistry and maxillofacial surgery remains at an early stage, iPSCs show great promise for generating specialized osteoblasts for craniofacial bone defect repair and potentially for future whole-tooth bioengineering. Current research focuses on developing safe differentiation protocols and effective delivery systems while minimizing the risk of teratoma formation, which is a major challenge associated with pluripotent cells [99].

The significance of stem cells in dentistry is particularly evident in the regeneration of alveolar bone following extractions, sinus augmentation, treatment of periodontal diseases, and implantology, where the provision of stable and well-vascularized bone tissue is essential for the success of dental implants. By combining MSCs, DPSCs, PDLSCs, ADSCs, and, in the future, iPSCs with advanced polymeric and bioactive scaffolds, it becomes possible to create an environment that supports functional tissue regeneration in a manner closely resembling physiological osteogenesis. This approach opens the prospect of achieving more predictable and long-lasting therapeutic outcomes in regenerative dentistry. Although still at an early translational stage, iPSC-based strategies remain one of the most promising frontiers in regenerative medicine, offering the potential to generate personalized, fully functional bone or dental tissues for future clinical applications.

#### 5.1.2. Vascularization and Oxygen Regulation

One of the main translational limitations in maxillofacial bone regeneration is the inability to achieve rapid and stable vascularization within large constructs. Diffusion of oxygen and nutrients is effective only within a radius of 100–200 μm, which means that scaffolds thicker than 3–5 mm undergo central necrosis unless a functional microvascular network forms early after implantation. This “vascularization bottleneck” is a major reason why many large grafts fail despite promising osteogenic results in small-animal models [100].

Prevascularization strategies attempt to address this limitation. Co-culture systems combining Endothelial Colony-Forming Cells (ECFCs), Human Umbilical Vein Endothelial Cells (HUVECs), and MSCs generate microvascular networks that can inosculate with host vessels within days of implantation. These preformed capillary beds significantly improve perfusion, osteogenesis, and long-term survival of engineered bone in large defects [101]. An alternative approach involves introducing microfluidic channels or sacrificial templates (e.g., Pluronic F127) into scaffolds to create perfusable lumens that guide angiogenic sprouting and accelerate inosculation [102].

Dynamic perfusion bioreactors further enhance vascular maturation by providing controlled flow, shear stress, and oxygenation. Perfused constructs show higher endothelial alignment, more robust ECM deposition, and superior bone formation compared to static cultures [103]. These systems are particularly relevant for maxillofacial reconstruction, where large, geometrically complex defects require rapid vascular integration to prevent graft failure.

Effective vascularization is closely linked to oxygen tension within engineered tissues. Bone regeneration requires tightly regulated oxygen levels: excessive hypoxia (<1–2% O_2_) triggers cell apoptosis and necrosis, while supraphysiologic oxygen tension disrupts angiogenic signaling and ECM mineralization. Large scaffolds often develop steep oxygen gradients, with hypoxic cores and better-oxygenated peripheries, leading to heterogeneous tissue quality and inadequate integration [104]. Strategies such as controlled hypoxia preconditioning of MSCs, oxygen-releasing microparticles, and perfused bioreactors help stabilize oxygen distribution and maintain metabolic homeostasis during early stages of regeneration, thereby supporting uniform osteogenesis across the entire scaffold volume.

### 5.2. Organoid-Based Approaches and 3D Bioprinting

The direction of modern reconstructive surgery is inevitably moving toward maximal personalization of treatment, in which traditional approaches based on synthetic materials are being replaced by biologically integrated solutions. The key technologies enabling this breakthrough are organoids and 3D bioprinting—innovative platforms that elevate tissue engineering from experimental laboratory models to clinically applicable therapies. Both technologies share a common goal: the creation of autologous, functional, and fully integrated tissue structures capable of regenerating complex systems such as bone, gingiva, or teeth.

Organoids are three-dimensional, self-organizing structures cultured in vitro that faithfully recapitulate the morphology, cellular complexity, and function of selected tissues or organs. They are derived from pluripotent stem cells—either embryonic or induced—which, under appropriate biochemical and mechanical conditions, spontaneously organize into tissue-like structures [105]. In the context of dentistry and craniofacial surgery, intensive research focuses on generating organoids derived from ectomesenchyme, including the dental follicle, dental papilla, and ameloblastic epithelium. These structures may serve as the foundation for future biological tooth regeneration—known as bio-tooth engineering [106]. Preliminary experiments conducted on animal models have demonstrated that dental organoids can initiate morphogenetic processes, including the formation of enamel and dentin primordia, as well as tissue mineralization [107]. Moreover, gingival and periodontal organoids show promising potential for research into soft tissue healing, implant integration, and periodontal disease therapy. The use of such structures could, in the future, enable the reconstruction of periodontal components in a manner far more biological than current regenerative techniques. Although organoids are presently used primarily as tools for studying disease pathogenesis, testing biomaterials, and personalizing pharmacological therapies, their translational potential for reconstructing stomatognathic structures is immense [108].

Three-dimensional bioprinting represents a groundbreaking technology that enables the fabrication of living, spatial tissue constructs through the layer-by-layer deposition of bioinks containing cells, growth factors, and biomaterials. Unlike traditional 3D printing, where the material is a synthetic polymer, bioprinting employs bioactive hydrogels—such as alginate, collagen, or gelatin methacrylate (GelMA)—laden with patient-derived cells [63].

The bioprinting process is based on medical imaging data, such as computed tomography (CT) or cone-beam computed tomography (CBCT), allowing the creation of an accurate three-dimensional model of a bone or soft tissue defect. Subsequently, a personalized scaffold is designed to match the patient’s anatomy precisely—not only in shape, but also in mechanical properties, porosity, and spatial distribution of biological cues [109].

#### 5.2.1. Bioink Standardization and Viscoelastic Challenges

A critical yet often underappreciated barrier to the clinical adoption of 3D bioprinting is the lack of standardized, reproducible, and functionally robust bioinks. Most current bioinks—including GelMA, alginate-based composites, and decellularized extracellular matrix (dECM) formulations are optimized primarily for printability rather than biological fidelity. GelMA offers tunable stiffness and excellent cytocompatibility but still lacks the hierarchical organization and mineral content characteristic of native bone ECM. Alginate-based systems provide favorable shear-thinning behavior and high print fidelity but require ionic crosslinking that limits mechanical strength and cell–matrix interactions. dECM bioinks more closely replicate tissue-specific biochemical cues, yet they suffer from weak mechanics, batch-to-batch variability, and inconsistent rheological properties [110].

The viscoelasticity of bioinks plays a central role in determining print fidelity, cell viability, and long-term structural stability of printed constructs. Effective bioinks must exhibit shear-thinning behavior—low viscosity under printing pressure to protect encapsulated cells—while rapidly recovering viscosity post-extrusion to preserve shape. However, achieving the optimal balance between printability and biological relevance remains difficult [111]. Many bone-targeted bioinks are simply too soft (1–10 kPa) compared to native bone ECM (hundreds of MPa to several GPa), resulting in insufficient mechanical cues for osteogenic differentiation and poor load-bearing capacity after implantation [112]. These discrepancies highlight the need for hybrid systems that integrate soft, cell-friendly hydrogels with stiffer ceramic or polymer frameworks to approximate the multiscale mechanical environment of bone.

Crosslinking strategies further complicate standardization. Photo-crosslinkable GelMA allows precise spatial control but depends on light penetration and photoinitiator distribution, which may reduce cell viability or create gradients in mechanical stiffness. Ionic crosslinking in alginate systems is rapid but prone to inhomogeneity and unpredictable long-term stability [113]. Enzyme-mediated crosslinking offers more physiological conditions yet remains slower and more difficult to control. As a result, reproducible tuning of viscosity, modulus, degradation rate, and cell responsiveness is challenging, limiting the scalability of bioinks for clinical manufacturing [114].

Collectively, these issues underscore the pressing need to establish standardized rheological benchmarks—such as yield stress, storage modulus, shear-thinning index, and crosslinking kinetics—to ensure reproducible printing outcomes across laboratories. Advances in composite bioinks (GelMA-ceramic hybrids, alginate-nanoclay systems, PCL-reinforced hydrogels) and dECM-based formulations supplemented with mineral nanoparticles represent promising attempts to bridge the gap between printability and biological fidelity [115,116]. However, significant progress is still required before bone-specific bioinks can fully replicate the mechanical, biochemical, and architectural complexity of the native maxillofacial microenvironment.

#### 5.2.2. Decellularized Extracellular Matrix (dECM) Biomaterials

Decellularized extracellular matrix (dECM) has emerged as a highly promising class of biomaterials for maxillofacial tissue engineering due to its ability to preserve the biochemical complexity, hierarchical architecture, and bioactive signaling cues of native tissues. dECM can be derived from bone, cartilage, dermis, dental pulp, periodontal ligament, or other connective tissues, providing tissue-specific guidance that supports cell adhesion, osteogenic differentiation, and angiogenesis. Unlike synthetic scaffolds, dECM retains native collagens, glycosaminoglycans, proteoglycans, growth factors, and matricellular proteins, which collectively create a microenvironment resembling natural healing conditions with inherently low immunogenicity [117,118].

Bone-derived dECM promotes osteogenesis through biomimetic presentation of type I collagen and non-collagenous proteins, while cartilage-derived dECM provides a favorable milieu for chondrogenic repair in mandibular condyle reconstruction. Dental pulp and periodontal ligament dECM support odontogenic and ligamentous lineage commitment, offering translational potential for periodontics and endodontic regeneration. These materials can be processed into hydrogels, micronized powders, coatings, or composite scaffolds, facilitating integration with 3D bioprinting and hybrid polymer–ceramic constructs [119].

Despite their advantages, dECM systems face important limitations. Donor-to-donor variability, incomplete decellularization, and batch inconsistency reduce reproducibility and complicate regulatory approval. Mechanical weakness, particularly in soft dECM hydrogels, often requires reinforcement with synthetic polymers or ceramic particles to achieve clinically relevant strength. Lack of standardized decellularization protocols and quality-control criteria remains a major barrier to widespread clinical translation [120]. Nonetheless, ongoing advances in tissue-specific dECM processing and crosslinking are rapidly improving their reliability, making dECM a key emerging biomaterial for next-generation maxillofacial regeneration.

## 6. Interactions Between Biomaterials and Host Biology

### 6.1. Bioactive Factors and Delivery Systems in Dental Therapy

To achieve optimal tissue regeneration—particularly in the craniofacial region—it is essential to ensure controlled and localized delivery of bioactive substances that modulate the cellular microenvironment and guide reparative processes. Central to this are bioactive factors—proteins, peptides, and signaling molecules responsible for regulating cell proliferation, differentiation, and migration, as well as for initiating angiogenesis and osteogenesis [121]. In clinical practice, these factors are often delivered directly to the defect site, commonly in the form of autologous concentrates such as platelet-rich plasma (PRP) or platelet-rich fibrin (PRF), which serve as natural reservoirs of multiple growth factors. Alternatively, these molecules can be incorporated into biomaterial structures, enabling their gradual and controlled release [122].

Among the most important biological regulators of regenerative processes are cytokines, which mediate intercellular communication and regulate inflammatory responses. Another key group is BMPs, with recombinant human BMP-2 (rhBMP-2) being particularly significant in dentistry [123]. This molecule exhibits exceptionally strong osteoinductive activity, stimulating MSCs differentiation into osteoblasts and initiating new bone formation. VEGF plays a pivotal role in angiogenesis, while PDGF regulates chemotaxis, proliferation, and differentiation of progenitor cells. Exosomes, nanoscale extracellular vesicles, carry microRNAs, proteins, and other signaling molecules capable of modulating gene expression in target cells, promoting osteogenesis, angiogenesis, and soft tissue regeneration without immunogenic risk [124].

The use of controlled-release systems in dentistry and maxillofacial surgery brings tangible benefits. BMP-2- and VEGF-enriched biomaterials, such as hydroxyapatite-based scaffolds, accelerate osseointegration and healing of bone defects. PDGF and exosomes promote regeneration of the periodontium, especially when combined with barrier membranes used in guided tissue regeneration (GTR) [1]. A summary of controlled release strategies for bioactive molecules applied in maxillofacial reconstruction, together with their mechanisms and therapeutic roles, is presented in Table 2.

### 6.2. Biomaterial–Tissue Interactions

The integration of a biomaterial with the surrounding tissue constitutes one of the key factors determining the success of regenerative and implant-based therapies. The performance of scaffolds and implants depends not only on the material itself but, to a large extent, on their ability to actively interact with host cells—including adhesion, proliferation, differentiation, migration, and modulation of the immune response. In recent years, considerable attention has been devoted to the design of biomaterials that, through their mechanical, topographical, and immunomodulatory properties, actively support regenerative processes and promote integration with the host tissue.

#### 6.2.1. The Role of Micro- and Nanostructures on Scaffold Surfaces

The surface topography of biomedical materials, including the presence of micro- and nanostructures, strongly influences cell behavior and directs their differentiation. Multiple studies have shown that the height and shape of nanocolumns or nanopillars (high aspect ratio of height to diameter) on titanium surfaces can regulate cell adhesion, morphology (contractility), and the nuclear translocation of transcription factors such as YAP and Runx2, thereby promoting osteogenic differentiation of MSCs. This mechanism is often dependent on the activity of focal adhesion kinase (FAK) and Rho-associated kinase (ROCK), which mediate the conversion of mechanical forces into intracellular signals, affecting the cytoskeleton and cell shape. For instance, studies on black titanium nanopillars (bTi) demonstrated that increased Runx2 expression depends on FAK and ROCK activity and YAP nuclear translocation, indicating the translation of mechanical cues into genetic signals that favor osteogenesis [117,118].

Nanostructures can also affect cell adhesion by regulating the number of focal adhesions and modulating cell stiffness, which serves as a mechanosensory function. This can lead to enhanced endocytosis of integrin receptors or cytoskeletal reorganization, indirectly modifying mechanotransduction pathways and activating osteogenic genes [119]. Additionally, nanoporous and microtopographical features facilitate the adsorption of extracellular matrix proteins, which is critical for initial cell attachment and the initiation of integrative biological processes. For instance, nanoporous titanium surfaces enhance mitochondrial oxidative phosphorylation and activate Piezo1 channels, improving osteointegration in vivo [120].

In animal studies examining modified implant surfaces, implants coated with micro- and nanostructures exhibited significantly higher bone-to-implant contact (BIC) than smooth implants. In one ovine study, implants with modified surfaces achieved a BIC of approximately 79–86%, compared to 68–75% for control implants after the same healing period [121].

#### 6.2.2. Immunomodulatory Biomaterials

**Figure 3:**
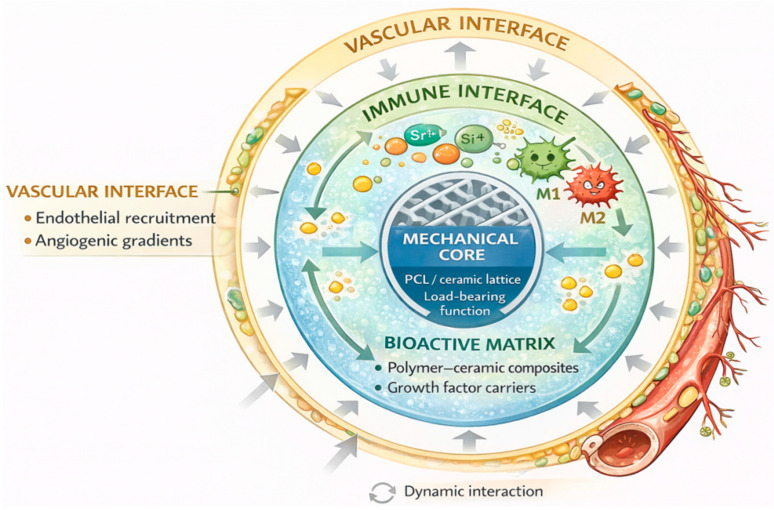
Integrated smart and biohybrid biomaterial systems for functional tissue regeneration.

Traditionally, the immune response to implants was viewed as an obstacle—a “hostile” reaction that could lead to fibrous encapsulation, inflammation, or improper integration. Modern approaches, however, recognize that this response can be harnessed constructively through immunomodulatory biomaterials, which actively influence immune cell behavior—particularly macrophages—toward a pro-regenerative phenotype. M1 macrophages are associated with a pro-inflammatory response, whereas the M2 phenotype promotes tissue repair and regeneration (Figure 3) [122].

Immunomodulatory biomaterials can influence macrophage polarization via controlled release of cytokines (e.g., IL-4, IL-10) or through specific modifications of surface topography, stiffness, and hydrophilicity, which encourage the shift toward the M2 phenotype. Literature reports describe implants coated with IL-4, which stimulated the expression of M2 markers (e.g., arginase-1), reduced inflammation, and limited the formation of thick fibrous capsules in murine models [123].

Reviews on immunomodulatory biomaterials emphasize that surface modifications—such as nanoscale structures, variable surface tension, functional groups (amine, carboxyl), or immobilized biomolecules (immunoregulatory ligands)—can direct macrophage activity, reduce foreign body giant cell (FBGC) formation, and promote an M2 regenerative phenotype [124].

Assessing the immune response is also critical in the design of bone-targeted biomaterials, as chronic activation of M1 macrophages can lead to excessive bone resorption and impaired implant integration. In peri-implant models, macrophages play a central role in regulating inflammation and tissue healing, particularly in the presence of bacterial biofilms. Their phenotype and activity can determine whether the local environment favors regenerative osteogenesis or pathological bone loss, highlighting the importance of immunomodulatory strategies in implant design [125].

#### 6.2.3. Microbiome-Osteoimmune Crosstalk in Maxillofacial Healing

Recent studies highlight that the oral microbiome plays a direct regulatory role in osteoimmune responses, influencing macrophage phenotype, osteoclast activity, and overall healing dynamics. Dysbiosis—particularly in peri-implant environments—shifts the local immune microenvironment toward a pro-inflammatory state dominated by M1 macrophages and elevated levels of TNF-α, IL-1β, and RANKL. This phenotypic bias disrupts early pro-regenerative signaling, delays resolution of inflammation, and promotes osteoclast-mediated bone resorption, contributing to implant instability and peri-implantitis. Conversely, a balanced microbiome supports M2 polarization through microbial metabolites (such as SCFAs), pattern-recognition receptor modulation, and reduced TLR-driven inflammatory cascades. This promotes pro-healing cytokine profiles, angiogenesis, and controlled remodeling of extracellular matrix. Several in vivo models demonstrate that specific microbial communities can enhance osteogenic differentiation of MSCs indirectly by shaping macrophage phenotype and reducing chronic low-grade inflammation at the implant–tissue interface [126,127,128,129].

These findings underscore the need to consider the microbiome as an integral component of osteoimmunology. Biomaterials with antibacterial or immunomodulatory coatings, probiotics, and microbiome-informed perioperative protocols are increasingly viewed as strategies to restore oral ecological balance and promote M2-driven regeneration. Integrating microbiome–immune axis insights into biomaterial design may help reduce peri-implant dysbiosis and improve long-term stability of maxillofacial reconstructions [130,131].

Macrophages orchestrate the transition from inflammation to regeneration, but their phenotype is strongly influenced by the initial neutrophil-driven cytokine milieu. Persistent release of IL-8, TNF-α, and reactive oxygen species delays M2 polarization and enhances recruitment of osteoclast precursors [132,133]. Conversely, timely resolution of neutrophil activity supports macrophage transition toward an M2 phenotype associated with secretion of IL-10, TGF-β, and pro-angiogenic factors such as VEGF, facilitating vascular ingrowth and subsequent osteoblast recruitment. This interconnected neutrophil–macrophage–osteoclast axis is further modulated by the oral microbio me: biofilm-associated pathogens amplify neutrophil activation and NET formation, leading to a sustained pro-osteoclastic state characteristic of peri-implantitis and chronic bone loss [134,135]. Understanding and therapeutically targeting this early innate immune triad—through biomaterial surface engineering, immunomodulatory coatings, or microbiome-informed interventions—may substantially improve the predictability of maxillofacial bone regeneration and long-term implant stability.

## 7. Future Directions

By categorizing biomaterials not only by composition but also by biological function and translational readiness, the proposed framework provides a practical roadmap for next-generation biomaterial design. This approach facilitates rational material selection based on clinical context, defect complexity, and regulatory feasibility, rather than relying solely on laboratory performance.

### 7.1. Critical Translational Bottlenecks in Maxillofacial Regenerative Strategies

**Figure 4:**
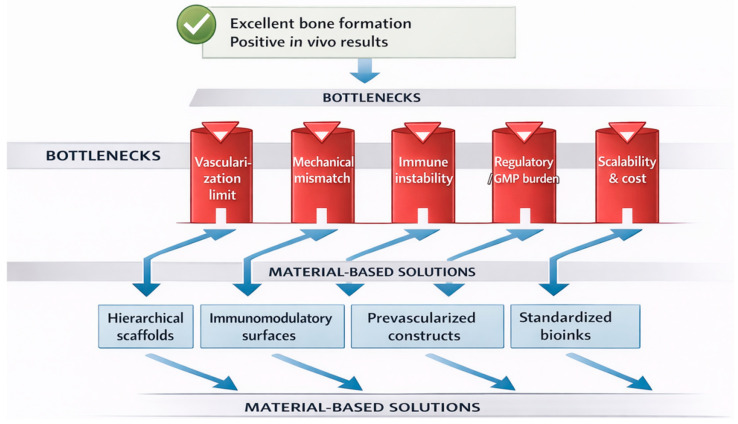
Translational bottlenecks and material-based solutions in craniofacial tissue engineering.

Despite substantial progress in biomaterials engineering and regenerative biology, several critical bottlenecks continue to limit the clinical translation of advanced maxillofacial regenerative strategies. However, promising preclinical outcomes do not necessarily translate into clinical success due to several persistent bottlenecks (Figure 4).

Vascularization remains the principal biological limitation, particularly for large and complex craniofacial defects. Diffusion constraints restrict oxygen and nutrient transport beyond 100–200 μm, resulting in central necrosis in thick scaffolds. Although prevascularization strategies, microfluidic architectures, and perfusion bioreactors show promise, their clinical scalability remains limited. Mechanical mismatch between engineered constructs and native craniofacial bone represents another major challenge. Many cell-laden hydrogels and bioinks exhibit elastic moduli several orders of magnitude lower than native bone, compromising load-bearing capacity and long-term stability after implantation. Standardization and regulatory classification constitute significant non-biological barriers. Cell-based therapies, dECM materials, and biohybrid constructs often fall under advanced therapy medicinal products (ATMPs), requiring GMP-compliant manufacturing, batch reproducibility, and extensive safety testing. Lack of harmonized regulatory frameworks slows clinical adoption. Biological variability and immunological unpredictability further complicate translation. Donor-dependent variability in MSCs, dECM composition, and exosome cargo affects reproducibility, while insufficient immunomodulatory control may lead to chronic inflammation or fibrotic encapsulation. Finally, cost, scalability, and clinical workflow integration remain underappreciated constraints. Technologies such as organoids and 3D bioprinting require specialized infrastructure and expertise, limiting their accessibility in routine clinical practice.

Addressing these bottlenecks will require interdisciplinary strategies integrating materials science, vascular biology, immunology, regulatory science, and digital manufacturing to enable predictable, scalable, and clinically viable regenerative solutions.

It is important to distinguish between evidence derived from preclinical and clinical studies when assessing the translational potential of biomaterials for craniofacial regeneration. The majority of advanced strategies discussed in this review—including ion-doped ceramics, smart polymers, cell-laden constructs, and biohybrid systems—are supported predominantly by in vitro experiments and small- or large-animal models. While these preclinical studies provide critical insights into osteogenic mechanisms, angiogenic responses, and immunomodulatory effects, their predictive value for clinical success remains limited by species-specific healing patterns, defect size discrepancies, and simplified loading conditions. In contrast, clinical evidence is currently restricted to a narrower range of commercially available materials, such as calcium phosphate ceramics, bioactive glasses, collagen-based carriers, and selected growth factor delivery systems, which demonstrate reliable osteoconductive performance but comparatively limited biological complexity. Bridging this gap requires not only biological efficacy but also regulatory compliance, manufacturing scalability, and reproducibility. Explicit differentiation between preclinical promise and clinically validated solutions is therefore essential for realistic assessment of translational readiness and for guiding future material development toward clinically implementable craniofacial regenerative therapies.

### 7.2. Strategies to Overcome Translational Barriers and Enable Clinical Adoption

Addressing the translational bottlenecks outlined above requires coordinated advances across biomaterials design, biological engineering, regulatory science, and digital manufacturing. To overcome vascularization limitations, hybrid strategies integrating prevascularized constructs with angiogenic factor gradients and perfusable architectures are gaining increasing attention. The combination of endothelial–mesenchymal co-culture systems, microfluidic channeling, and controlled VEGF delivery represents a promising route toward rapid inosculation and sustained perfusion in large craniofacial constructs. Importantly, these approaches are being progressively adapted to clinically scalable fabrication workflows. Mechanical mismatch and insufficient load-bearing capacity may be addressed through hierarchical scaffold design, in which stiff, load-bearing frameworks (e.g., PCL, titanium, or ceramic lattices) are combined with soft, cell-laden hydrogels. Such multiscale constructs better replicate the mechanical heterogeneity of native bone while preserving cell viability and biological functionality. Advances in multi-material 3D printing enable precise spatial control over stiffness gradients, enhancing both structural integrity and osteogenic signaling. Standardization and regulatory complexity remain major non-biological barriers. Progress toward GMP-compatible manufacturing of bioactive scaffolds, cell therapies, and extracellular vesicle-based products is essential. Establishing standardized bioink formulations, decellularization protocols, and quality-control benchmarks (e.g., rheological, biochemical, and immunological parameters) will improve reproducibility and facilitate regulatory approval. Early dialog with regulatory agencies and alignment with emerging ATMP frameworks are increasingly recognized as critical components of translational success. Biological variability and immune unpredictability may be mitigated through immunomodulatory biomaterials that actively direct host immune responses toward regenerative phenotypes. Surface-engineered scaffolds promoting M2 macrophage polarization, controlled cytokine delivery, and microbiome-aware antibacterial strategies offer promising routes to stabilize the local healing environment and reduce inter-patient variability. Finally, clinical integration and cost-effectiveness must be considered early in the design of regenerative technologies. AI-assisted digital planning, automated scaffold design, and streamlined manufacturing pipelines can reduce production costs and improve reproducibility. Integration of regenerative constructs into existing surgical workflows—rather than requiring entirely new infrastructure—ill be essential for widespread clinical adoption.

Collectively, these strategies highlight a shift from purely material-centric innovation toward system-level solutions that integrate biological performance, manufacturability, regulatory compliance, and clinical practicality.

### 7.3. AI-Assisted and Data-Driven Biomaterial Design

Artificial intelligence (AI) and data-driven design approaches are emerging as powerful tools in the development of next-generation biomaterials for craniofacial regeneration. Machine learning algorithms are increasingly used to optimize scaffold architecture, predict mechanical performance, and tailor porosity, stiffness gradients, and degradation profiles based on patient-specific imaging data. AI-assisted topology optimization enables the design of scaffolds that balance load-bearing capacity with biological permeability, addressing one of the key challenges in maxillofacial reconstruction.

Moreover, predictive models integrating material composition, cellular responses, and clinical outcomes offer new opportunities to accelerate biomaterial screening and reduce reliance on extensive animal testing. When combined with additive manufacturing and digital surgical planning, AI-driven design pipelines support truly personalized regenerative solutions, aligning material properties with anatomical, biomechanical, and biological requirements. Although still in an early translational phase, AI-assisted biomaterial design represents a promising direction toward more efficient, reproducible, and clinically scalable craniofacial regenerative therapies.

## 8. Conclusions

Advances in regenerative medicine, smart biomaterials, and digital technologies are reshaping the field of maxillofacial surgery, moving it toward increasingly personalized and biologically integrated therapeutic strategies. Although significant progress has been made in scaffold engineering, bioactive ceramics, and autologous biologics, only a limited subset of innovations has achieved broad clinical translation, while cell-based therapies, organoids, and 3D bioprinting remain largely experimental. Their adoption is constrained by vascularization challenges, biological variability, mechanical limitations, and complex regulatory pathways.

At the same time, digital planning, AI-supported diagnostics, and robotic-assisted procedures offer new levels of precision and standardization, with the potential to reduce complications and improve functional outcomes. The integration of intelligent materials, controlled drug-release systems, and biohybrid constructs may ultimately shift bone and soft-tissue reconstruction from passive defect replacement to active, adaptive regeneration that more closely mimics natural healing.

Future progress will depend on the harmonization of regulatory standards, improvements in manufacturing reproducibility, and the development of clinically validated workflows that ensure both safety and accessibility. As sustainability and ethical considerations become increasingly prominent, the evolution of maxillofacial regenerative strategies will require balancing technological advancement with responsible implementation. Together, these developments indicate a future in which biological effectiveness, technological precision, and patient-centered design converge to expand therapeutic possibilities and improve long-term outcomes.

## Figures and Tables

**Table 1:** Summary of Key Biomaterials Used in Maxillofacial Reconstruction.

Material/Class	Biological Target	Evidence Level	Clinical Use	Translational Readiness	Limitations	Key Advantages/Translational Releva
HA/TCP/BCP ceramics	Osteoconduction	Clinical (RCTs, cohorts)	Yes	High	Brittle; limited for load-bearing; limited bioactivity	Clinical familiarity; regulatory approval; predictable bone regeneration
Ion-doped ceramics	Enhanced bioactivity	In vivo (large animals)	Strong preclinical	Medium	Variable degradation; regulatory approval, dose control	Tunable osteogenic and angiogenic activity; enhanced biological performance
Polymers (PLGA, PCL, PEG)	Controlled degradation, drug delivery, soft-tissue support	In vivo + early clinical	Limited	Medium/High	Low intrinsic bioactivity	Processability; scalable manufacturing; versatile drug delivery platforms
Smart materials	Stimuli-responsive, controlled release, immunomodulation	In vitro/small animals	No	Low/Medium	Mechanical weakness	Spatiotemporal control of biological cues; adaptive microenvironment modulation
MSC/DPSC-based constructs	Cellular regeneration	Early clinical trials	Limited	Medium	GMP, donor variability	Strong regenerative potential; paracrine and immunomodulatory effects
iPSC-derived osteogenic cells	Personalized regeneration	In vitro/preclinical	No	Low	Safety, tumorigenicity	Patient-specific therapies; high differentiation potential
Three-dimensional bioprinting (bioinks)	Patient-specific constructs	Preclinical	No	Low	Vascularization, standardization	Anatomical customization; integration with digital surgical planning
dECM-based scaffolds	ECM mimicry	In vitro	No	Medium	Batch variability, weak mechanics	Biomimetic biochemical cues; enhanced cell–matrix interactions

**Table 2:** Controlled Release Strategies for Bioactive Molecules in Maxillofacial Reconstruction.

Bioactive Factor	Delivery System	Release Profile	Biological Role	Limitations	Key Advantages/Clinical Potential
BMP-2	PLGA microparticles; collagen sponges; hydrogels	Burst + sustained	Osteoinduction	Risk of ectopic bone; dose sensitivity	Strong osteoinductive efficacy; established clinical precedent
VEGF	Hydrogels; nanoparticle carriers; layered constructs	Fast or sequential	Angiogenesis	Short half-life; requires co-delivery	Rapid vascularization; critical for large defect integration
PDGF	Microspheres; electrospun fibers	Sustained	Cell recruitment + proliferation	Limited duration	Clinically validated signaling; enhanced early-stage healing
Exosomes	Hydrogels; EV-loaded fibers	Sustained low dose	Immunomodulation + angiogenesis	Standardization challenges	Cell-free therapy; reduced immunogenicity and safety risks
Antimicrobials	Dual-release coatings; Ag/Cu nanoparticles	Immediate + sustained	Infection control	Cytotoxicity risk	Localized antimicrobial protection; reduced systemic antibiotic use

## Data Availability

No new data were created or analyzed in this study. Data sharing is not applicable to this article.

## Abbreviations

The following abbreviations are used in this manuscript:

ADSCs	Adipose-Derived Stem Cells
ALP	Alkaline Phosphatase
BAG	Bioactive Glasses
BCPs	Biphasic Calcium Phosphates
BIC	Bone-To-Implant Contact
BMP-2	Bone Morphogenetic Proteins
CBCT	Cone-Beam Computed Tomography
CuNP	Copper Nanoparticles
dECM	Decellularized Extracellular Matrix
DPSCs	Dental Pulp Stem Cells
ECFCs	Endothelial Colony-Forming Cells
ECM	Extracellular Matrix
FAK	Focal Adhesion Kinase
FBGC	Foreign Body Giant Cell
GelMA	Gelatin Methacryloyl
HA	Hydroxyapatite
HUVECs	Human Umbilical Vein Endothelial Cells
iPSCs	Induced Pluripotent Stem Cells
MMPs	Matrix Metalloproteinases
MSCs	Mesenchymal Stem Cells
OCN	Osteocalcin
OPG	Osteoprotegerin
PCL	Poly-Ε-Caprolactone
PDGF	Platelet-Derived Growth Factor
PDLSCs	Periodontal Ligament Stem Cells
PLGA	Poly(Lactic-Co-Glycolic Acid)
PRF	Platelet-Rich Fibrin
PRP	Platelet-Rich Plasma
RANK	Receptor Activator Of Nuclear Factor
RGD	(Arginine–Glycine–Aspartic Acid) Sequence
ROCK	Rho-Associated Kinase
ROS	Reactive Oxygen Species
Runx2	Runt-Related Transcription Factor 2
SMPs	Shape-Memory Polymers
TGF-β	Transforming Growth Factor Β
VEGF	Vascular Endothelial Growth Factor
β-TCP	Β-Tricalcium Phosphate
